# *In vitro* biocompatibility analysis of protein-resistant amphiphilic polysulfobetaines as coatings for surgical implants in contact with complex body fluids

**DOI:** 10.3389/fbioe.2024.1403654

**Published:** 2024-07-17

**Authors:** Jana F. Karthäuser, Dierk Gruhn, Alejandro Martínez Guajardo, Regina Kopecz, Nina Babel, Ulrik Stervbo, André Laschewsky, Richard Viebahn, Jochen Salber, Axel Rosenhahn

**Affiliations:** ^1^ Analytical Chemistry—Biointerfaces, Ruhr University Bochum, Bochum, Germany; ^2^ Experimental Surgery, Ruhr University Bochum, Bochum, Germany; ^3^ Department of Surgery, Knappschaftskrankenhaus Bochum, University Hospital of the Ruhr University Bochum, Bochum, Germany; ^4^ Institute of Chemistry, Universität Potsdam, Potsdam, Germany; ^5^ Centre for Translational Medicine, Medical Department I, Marien Hospital Herne, University Hospital of the Ruhr University Bochum, Herne, Germany; ^6^ Fraunhofer Institute of Applied Polymer Research IAP, Potsdam, Germany

**Keywords:** polyzwitterion, antiadhesive, hydrogels, benzophenone methacrylate, platelet adhesion, thrombocyte activation

## Abstract

The fouling resistance of zwitterionic coatings is conventionally explained by the strong hydrophilicity of such polymers. Here, the *in vitro* biocompatibility of a set of systematically varied amphiphilic, zwitterionic copolymers is investigated. Photocrosslinkable, amphiphilic copolymers containing hydrophilic sulfobetaine methacrylate (SPe) and butyl methacrylate (BMA) were systematically synthesized in different ratios (50:50, 70:30, and 90:10) with a fixed content of photo-crosslinker by free radical copolymerization. The copolymers were spin-coated onto substrates and subsequently photocured by UV irradiation. Pure pBMA and pSPe as well as the prepared amphiphilic copolymers showed BMA content-dependent wettability in the dry state, but overall hydrophilic properties *a fortiori* in aqueous conditions. All polysulfobetaine-containing copolymers showed high resistance against non-specific adsorption (NSA) of proteins, platelet adhesion, thrombocyte activation, and bacterial accumulation. In some cases, the amphiphilic coatings even outperformed the purely hydrophilic pSPe coatings.

## Graphical Abstract

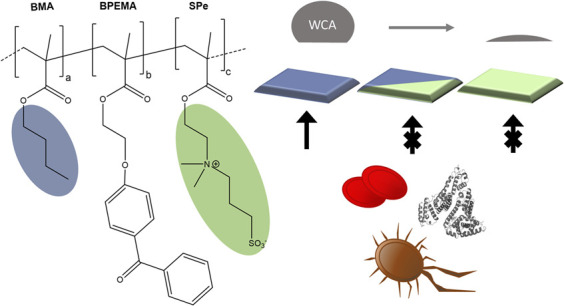

## 1 Introduction

Central venous or arterial catheters in permanent blood contact, chest drains, drainage tubes for draining bile, pancreatic secretions, urine or for draining wound fluids, or stents for keeping the ureters open and many other such medical devices have become indispensable in modern surgery or interventional medicine. (Maki et al., 2006; Durai et al., 2009; Safdar et al., 2013; Feneley et al., 2015). Tubular systems used for the transport of body fluids are mostly based on non-biodegradable polymeric biomaterials (e.g., silicones, polyurethanes, etc.) (Shastri, 2003) and only function adequately if their intraluminal diameter remains unchanged and is not constricted or even closed by protein/cell deposits (Baier, 2006), clotted blood (Suojanen et al., 2000), encrustations, (Tomer et al., 2021), or as a result of bacterial adhesion and biofilm formation. (Veerachamy et al., 2014). In order to prevent these adverse effects on the one hand, and to avoid triggering acute or chronic toxic or allergic reactions on the other, high demands are placed on biocompatible coatings for biomedical applications. By preventing non-specific adsorption (NSA) of proteins, the non-specific adhesion of human cells and other microorganisms can also be minimized. This improves hemocompatibility and reduces unwanted inflammatory or immune reactions.

The common approach is to use hydrophilic coatings such as polyethylene glycol-based materials because they are highly protein resistant due to their hydrophilic nature. However, the chemical nature of the polyethylene glycol (PEG) structure is prone to oxidation (Chapman et al., 2000; Jiang and Cao, 2010; Zheng et al., 2017), has shown in some instances to be immunogenic (Li et al., 2018; 2019), and provides little opportunity for chemical variations. Another popular class of hydrophilic polymers contains zwitterionic functional groups. (Jiang and Cao, 2010; Zheng et al., 2017; Blackman et al., 2019; Paschke and Lienkamp, 2020). Zwitterions are inspired by membranes of mammalian cells, which bear phospholipids and glycoproteins. (He et al., 2016). Coatings based on phosphatidylcholines (PC), carboxybetaines (CB), sulfobetaines (SB), or sulfabetaines (SAB) incorporate negatively and positively charged groups, which induce strong electrostatic interactions with surrounding water molecules, while the overall charge is neutral. (Lowe and McCormick, 2002; Jiang and Cao, 2010; Mi and Jiang, 2014; Venault and Chang, 2019). The type of functional groups, the orientation of the charged groups within the coating, and the overall architecture of the zwitterions enable a great variety of design and adaption flexibility for low fouling applications. (Baggerman et al., 2019; Blackman et al., 2019; Laschewsky and Rosenhahn, 2019). Common applications of zwitterion-containing coatings are the usage in biosensors (Yang et al., 2011; Wu et al., 2018) as well as for membranes. (Chang et al., 2011; Zhang et al., 2013; Liu et al., 2014; Duong et al., 2019). The addition of hydrophobic components into hydrophilic materials results in amphiphilic coatings which showed superior performance in several experiments. (Ishihara et al., 1992; Colak and Tew, 2012b; Lin et al., 2019; 2020; Wu et al., 2019; Dun et al., 2020). Polymers composed of PC and BMA exhibit a delayed coagulation time and reduced protein adsorption compared to polymers made solely of BMA. (Ishihara et al., 1992). Copolymers of ∼30% CB and ∼70% BMA showed high resistance against fibrinogen (Fb) adsorption. (Lin et al., 2019). In other experiments less systematic trends were observed, as the polybetaines combined with hydrophilic PEG or lipophobic fluorinated residues showed low NSA of proteins while polybetaines with lipophilic alkyl residues showed a higher NSA of proteins. (Colak and Tew, 2012a; Colak and Tew, 2012b).

The PC motif is known to improve the hemocompatibility of polymers. (Hayward and Chapman, 1984; Ishihara et al., 1990b; Ishihara et al., 1992; Ishihara et al., 1998; Nakaya and Li, 1999). For example, Ishihara et al. have co-polymerized 2-methacryloyloxyethyl phosphorylcholine (MPC) with butyl methacrylate (BMA) and demonstrated that increasing the MPC contents improved the non-thrombogenicity of the polymer. (Ishihara et al., 1992). Formulations based on this system with a molar PC content of about 30 mol% are nowadays commercially available as blood-compatible coatings for medical purposes (LIPIDURE-CM5206). (Ishihara et al., 1990a; 1990b; Ishihara, 2019). Similarly, CB functionalized monomers were copolymerized with BMA in different ratios to produce different amphiphilic coatings. Coatings with 30 mol% CB showed the best resistance against NSA of fibrinogen, and also demonstrated resistance against albumin, γ-globulin, and serum. Copolymers with more than 30 mol% CB units easily dissolved in an aqueous solution, and therefore, lost their ability to act as a protective coating. (Lin et al., 2019). In a different study, amphiphilic coatings containing 30% CB monomers were copolymerized with different hydrophobic components. All copolymers showed superior low fouling and thrombogenic response compared to uncoated materials. (Lee et al., 2021). To enhance the stability of zwitterionic polymers, Lin and co-workers co-polymerized MPC/BMA with photoreactive benzophenone-based methacrylate. The co-polymer was photo-crosslinked by UV radiation and showed high resistance against NSA of fibrinogen and HeLa cells. (Lin et al., 2015). Another study copolymerized methacrylate-based SB in combination with N-isopropylacrylamide (NIPAM) in different ratios. The higher the content of zwitterions in the resulting copolymers, the better the resistance to NSA of proteins, platelets, and human fibroblasts, while increased NIPAM content leads to improved thermosensitivity. (Chang et al., 2010). Still, despite these promising findings, several theoretical and experimental studies emphasize the view, that the ways of interaction of water molecules with different zwitterionic moieties vary substantially. (Shao and Jiang, 2014). According to these different modes of interactions, different levels of efficiency in their anti-fouling performance have been discussed. Mostly, PC-based coatings have been postulated to be the best, yet the experimental base for such comparisons is small. (Huang et al., 2018; Ishihara, 2019). PC-moieties pose the risk of hydrolytic cleavage in a biological environment. (Laschewsky and Rosenhahn, 2019). Comparing CB and SB moieties, both have a high hydration capacity but SB is less sensitive to different ions and pH changes and has still only a moderate self-assembling effect. (Chen and Jiang, 2008; Shao et al., 2010; 2011).

While the combination of PC-methacrylates with BMA showed promising properties, combinations of SB-methacrylates with BMA have not yet been explored regarding “*in vitro* biocompatibility.” We recently synthesized a series of photocrosslinkable coatings containing SPe and BMA components in different ratios and evaluated them in the context of marine fouling. Our results show that the effectiveness of these amphiphilic coatings varies significantly depending on the specific combination of copolymer and fouling organisms. (Schardt et al., 2022). In order to evaluate their utility as potential medical coating comprehensively, it is necessary to extend the studies with respect to resistance to biological cells and blood components. In the present work, the proportions of 3-(*N*-(2-methacryloyloxy)ethyl-*N,N*-dimethylammonio)-propane-1-sulfonate (SPe) and n-butyl methacrylate (BMA) were systematically varied from approximately 50, 70, and 90% SPe mixed with 50, 30% and 10% BMA and were compared to pure pSPe and pBMA. Each coating contained approx. 1 mol% of 2-(4-benzoylphenoxy) ethyl methacrylate (BPEMA) as a photoreactive crosslinker to enhance its stability. The produced homogeneous and well-defined SPe/BMA coatings with systematically varied hydrophobic content were analyzed regarding their long-term stability in phosphate-buffered saline (PBS) solution and their resistance to the NSA of human serum albumin (HSA) and fibronectin (Fn). Furthermore, the adhesion of L929 mouse fibroblasts and primary human thrombocytes as well as their aggregation and activation could be prevented. In addition, these coatings showed no hemolysis of erythrocytes nor leucocyte activation. Also, the prevention of *Escherichia coli* adhesion was shown to be a significant and beneficial property for practical clinical applications.

## 2 Materials and methods

### 2.1 Chemicals

2,2,2-trifluoroethanol (TFE, 99,8%, Carl Roth, Germany), 3-aminopropyltrimethoxysilane (APTMS, 97%, Sigma Aldrich, United States), 11-aminoundecane thiol hydrochloride (AUDT, ProChimia Surfaces, Poland), acetone (HPLC grade, Alfa Aesar, United States), ethanol (analytical reagent grade, Fisher Scientific, United States), hydroxyhexaethyleneglycol-1-undecanethiol (EG_6_OH, ProChimia Surfaces, Poland), albumin from human serum (HSA, lyophilized powder, 97%–99%, Sigma-Aldrich, United States), fibronectin from human plasma (Fn, lyophilized powder, ≥95%, Sigma-Aldrich, United States), 1-dodecanethiol (DDT, Sigma-Aldrich United States), 3-(*N*-(2-methacryloyloxy)ethy-*N,N*-dimethylammonio)propane-1-sulfonate (SPe, Sigma-Aldrich, ≥97%), 2-(4-Benzoylphenoxy)ethyl methacrylate (BPEMA, a gift from Michael Pach, IAP Fraunhofer Potsdam/Germany), Müller-Hinton-Boullion (Carl Roth, Karlsruhe, Germany), nutrient agar (Carl Roth, Karlsruhe, Germany), phosphate-buffered saline (10× PBS, pH = 7.4, Fisher Scientific, United States), and LIVE/DEAD™ BacLight™ (propidium iodide, SYTO 9 (Thermo Fisher Scientific, United States) were used without further purification. Butyl methacrylate (BMA, Sigma Aldrich, containing 10 ppm of monomethylhydroquinone “MEHQ” as an inhibitor) was distilled before used. Water was purified with a Milli-Q system (Siemens Water Technology, Germany). Coatings (∼100 nm thickness) were prepared on silicon wafers (Siegert Wafer, Germany) for thickness determination, on glass (Nexterion Slide Glass B or D263, Schott, Germany) for biological experiments and captive bubble contact angle goniometry, or on gold-coated glass slides (Ti 5 nm + Au 60 nm) for protein resistance assessment by surface plasmon resonance spectroscopy.

### 2.2 Sample preparation

Silicon and glass substrates were modified with 3-aminopropyltrimethoxysilane (APTMS). Gold substrates were modified with a self-assembled monolayer of 11-amino-1-undecanethiol hydrochloride (AUDT) as adhesion-promoting layer according to previously established protocols. (Thome et al., 2012; Arpa Sancet et al., 2013; Bauer et al., 2016). All samples were stored under inert atmosphere until further use. Copolymerization of 2-(4-benzoylphenoxy)-ethyl methacrylate (**BPEMA**) with the monomers *n*-butyl methacrylate (**BMA**), and 3-[N-2-(methacryloyloxy)ethyl-N, N-dimethyl]-ammonio propane-1-sulfonate (**SPe**) was conducted in different ratios by the usage of free radical copolymerization as previously described. (Buller et al., 2013; Schönemann et al., 2018; Koc et al., 2019; Schardt et al., 2022). Molecular characterization of the co-polymers including ^1^H-NMR, size exclusion chromatography, and UV-VIS spectroscopy was previously reported. (Schardt et al., 2022). Co-polymers were dissolved in 2,2,2-trifluoroethanol (TFE), spin-coated (10 s at 100 rpm followed by 30 s at 3,000 rpm) onto the different substrates and photo-crosslinked by UV-irradiation [Dr. Hönle AG, UVA Cube 100 (100 W) with Strahler UV 150F (150W)] following published protocols. (Koc et al., 2019). Solutions of 1 wt% were used on pre-treated silicon and glass substrates for analytical characterization. Solutions of 0.5 wt% were applied on pre-treated gold substrates for protein resistance analysis. Co-polymerized compounds are indicated using the prefix **p** when exclusively BMA or SPe is used in combination with BPEMA or **cop** when BMA and SPe are used in different ratios in combination with BPEMA.

### 2.3 Contact angle goniometry

Static water contact angles (WCA) in air and captive bubble contact angles (CBCA) underwater were determined with two different custom-built angle goniometers as previously reported. (Koschitzki et al., 2020). For static water contact angle measurements, a droplet (∼15 µL) of tri-distilled water was applied and for underwater contact angle measurements air bubbles (∼10–15 µL) were applied from below to the coating facing downwards. The shapes of the air bubbles were analyzed according to previous protocols. (Koschitzki et al., 2020). For both techniques, each value represents the average of at least three measurements on three different samples (n = 9), and given error bars represent the standard deviation.

### 2.4 Spectroscopic ellipsometry

The thickness of the coatings was monitored by a multiple wavelength ellipsometer (M-2000, J. A. Woollam Co., United States) according to previously established protocols. (Koc et al., 2019; Gnanasampanthan et al., 2021). For modified surfaces with thicknesses below 60 nm, the surfaces were modeled as a single transparent layer with a wavelength-depending refractive index (Cauchy model: A = 1.45, B = 0.01, C = 0), and for modified surfaces with thickness above 60 nm, the surfaces were modeled as an absorbing layer. Values represent the average of at least three measurements on three different samples (n = 9) and given error bars represent the standard deviation.

### 2.5 Stability determination

The stability of the coatings was determined by under-water contact angle goniometry and spectroscopic ellipsometry. Coatings were immersed under dynamic conditions (linear shaker, 60 rpm) in 1 x PBS (pH = 7.4) for different durations. After 1 min, 10 min, 1 h, 3 h, 1 day, 2 days, and 7 days the captive bubble contact angles and thicknesses were determined. In addition, the sessile contact angle was determined, which indicates the value at the time 0 min. Each value represents the average of at least three measurements on three different samples (n = 9), and the error bars represent the standard deviation.

### 2.6 Surface plasmon resonance spectroscopy

Nonspecific protein adsorption of human serum albumin (HSA) and fibronectin (Fn) was determined using surface plasmon resonance spectroscopy (SRC7000DC, pump SR7500, autosampler SR7100, Reichert, United States) following previously established protocols. (Koc et al., 2019; Gnanasampanthan et al., 2021; Schönemann et al., 2021). After baseline equilibration, HSA solutions (1 mg/mL) were injected over a period of 10 min at a flow rate of 10 μL/min. For Fn measurements, a concentration of 0.05 mg/mL was injected with a higher flow rate of 20 μL/min over a period of 10 min. Subsequently, 0.5 x PBS was injected to determine the amount of reversibly bound protein. The amount of irreversibly bound protein was determined from the SPR signal difference before the injection of the protein and after a PBS injection phase of 20 min. Each value represents the average of at least three measurements, and reported error bars represent the standard deviation.

### 2.7 L929 mouse fibroblasts adhesion assay

Cells of the L929 murine fibroblast cell line were obtained from DSMZ (German Collection of Microorganisms and Cell Cultures). L929 cells maintained in cell culture media RPMI 1640 with stable glutamine (PAN Biotech, Germany) containing 10% fetal bovine serum (FBS; PAN Biotech, Germany) under physiological culture conditions (37°C, 5% CO_2_), and subcultured using 0.25% Trypsin (Gibco, United States). The glass substrates with the coatings to be analyzed had a diameter of 20 mm. The cell number/volume of the L929 mouse fibroblasts was 100,000/mL. From this initial suspension, 200 µL (approx. 20,000 cells) were pipetted onto the central area of each sample surface and incubated for 3 h under standard cultivation conditions. Subsequently, 1 mL of fresh complete medium was added without rinsing the previously applied suspension away from the surface. All samples to be analyzed were then incubated for a further 20 h. The next day, brightfield microscopic live imaging of the sample surfaces was carried out. A customized live cell imaging system using the Olympus microscope type IX51 (Olympus, Germany) was used for this purpose. After the samples were removed from the cell culture incubator, they were immediately transferred to the microscope incubator so that the behavior of the cells could be observed under ideal cultivation conditions. Tissue culture polystyrene (TCPS, pro-cell adhesion, positive control) and polystyrene (PS, for suspension culture, negative control) served as control surfaces. Three replicate experiments (n = 3) were performed for each sample surface.

### 2.8 Surface cytotoxicity assay

To determine the cytotoxic effects of the surface modifications on L929 (German Collection of Microorganisms and Cell Cultures) a membrane integrity assay was performed (CytoTox™-ONE Homogeneous Membrane Integrity Assay, Promega, United States). Glass cover slips (ø 18 mm) modified with sample surfaces were placed in a 12-well-plate and fixed using 12-well plate inserts (CellCrown™12, Scaffdex, Finland). Sample sterilization was done by UV irradiation (UVC, ∼254 nm, 15 W) for 30 min and afterwards washed twice with DPBS (Gibco, United States). L929 were obtained as described previously. Cells [100,000 cells in 1 mL RPMI 1640 with stable glutamine (PAN Biotech, Germany) with 10% FBS (PAN Biotech, Germany)] were preincubated on substrate surfaces and two TCPS controls for 1 h at physiological culture conditions (37°C, 5% CO_2_) and followed by addition of 1 mL medium. Samples were incubated for 48 h in advance of the assay. Assay reagents were prepare as described by the distributor. For maximum LDH (lactate dehydrogenase) release, cells of one TCPS control were lysed using 0.1% TritonX-100 (for mol. Biology, Sigma Aldrich, Germany) for 10 min. The assay was performed as reported in the manual. Fluorescence was measured at ex/em 560 nm/590 nm (CLARIOstar Plus, BMG LABTECH, Germany). The results are displayed as relative LDH release compared to maximum LDH release Eq. (1). Measurement was performed in triplicates (n = 3).

$$LDH\;Release\;\left[\%\right]=\frac{Measured\;LDH\;Release-Background}{Maximum\;LDH\;Release-Background}\times 100\%$$ (1)

### 2.9 Blood collection

Fresh human whole blood was obtained from healthy adult volunteers (no medication (e.g., aspirin) in the previous 2 weeks) by venipuncture into a S-Monovette 9 mL LH (Sarstedt, Nümbrecht, Germany). Blood was collected at the Centre for Translational Medicine, Medical Department I, University Hospital of the Ruhr-University Bochum, Herne, Germany according to appropriate legal and ethical guidelines. The participants provided written informed consent to participate in this study. The study was reviewed and approved by the Ethics Committee of the Medical Faculty of the Ruhr-University Bochum, Gesundheitscampus 33, D-44801 Bochum. The registration number of the study is 16-5,649.

### 2.10 Human thrombocytes adhesion assay

To perform the platelet adhesion assay, coverslips coated with the different polymers were placed individually into 6-well plates (one sample per well, PS). The samples were washed three times with 2 mL of a 0.9% (w/v) sodium chloride solution (B. Braun Melsungen, Germany) each, and shaken on a plate shaker for 1 min with 300 rpm at 37°C and dried. Fresh venous blood was collected via a peripheral arm vein as prescribed and transferred directly into lithium heparinate (LH) monovettes (S-Monovette 9 mL LH, SARSTEDT, Germany). The LH blood was centrifuged (5810 R, equipped with rotor A-4–62, Eppendorf, Germany) with 250 x g, 10 min at room temperature (RT). 200 μL of the clear phase (platelet-rich plasma, PRP) of the supernatant was diluted in 800 µL 0.9% (w/v) NaCl solution (1:4). 200 μL of the dilution were carefully placed on the glass coverslips. The samples were incubated for 1 h at 37°C at 200 rpm. Afterwards, the supernatant was removed, and the samples and controls were washed three times with 500 µL DPBS (GIBCO, Life Technologies, United Kingdom). The cells were fixed using 500 µL of a 4% (v/v) formaldehyde solution in DPBS for 30 min at RT. After the fixation solution was removed, the samples and controls were washed once with 500 µL DPBS each. Then 500 µL of a 0.4% (v/v) Triton X-100 solution (Molecular biology, Sigma-Aldrich, Germany) in DPBS was added to the cells for 10 min at RT. After disposal of the solution, the cells were washed once with 500 µL DPBS. The samples and controls were incubated for 1 h at RT in 500 µL of a 2% (w/v) BSA (≥96%, Sigma-Aldrich, Germany) solution in DPBS. The supernatant was discarded, and the cells stained with 200 µL of a 1:250 Phalloidin 546 (PromoFluor546 Labeled Phalloidin, PromoKine, Germany) in 2% (w/v) BSA solution per well. The cells were stained for 30 min at RT under light exclusion. Last, the samples were washed three times with 500 µL each of DPBS and dried. Fluorescence images were recorded using the Olympus IX51 microscope equipped with camera Olympus XM10 (Olympus, Germany) at Ex/Em = 551 nm/572 nm. A duplicate determination was carried out for each surface modification.

### 2.11 Whole blood adhesion assay

To analyze the adhesion of other blood components, the thrombocyte adhesion was repeated with whole blood. Two coverslips of each surface modification, including TCPS cover slips as control (ø 13 mm, Sarstedt, Germany) were placed inside wells of 6-well plates (one sample per well, PS). The different surface coatings and control were each washed once with 2 mL of a 0.9% (w/v) NaCl solution (B. Braun Melsungen, Germany) and shaken for 1 min at 300 rpm at 37°C. The samples were equilibrated in 2 mL of fresh NaCl solution for 30 min at 37°C prior incubation with undiluted LH blood for 1 h at 300 rpm and 37°C. The supernatant was removed, and the samples and controls were washed in DPBS (GIBCO, Life Technologies, United Kingdom) by dipping the cover slips several times. This was repeated two more times with fresh DPBS to remove unattached blood components. The cells were fixed and stained as described for thrombocyte adhesion. Microscopy was performed using the Olympus IX51 microscope equipped with camera Olympus XM10 (Olympus, Germany) at Ex/Em = 551 nm/572 nm.

### 2.12 Human erythrocytes-based dynamic hemolysis assay

To measure the hemolysis of human erythrocytes in contact with the polymer coatings, three samples of each surface modification were placed on coverslips in the wells of 6-well plates (one sample per well, PS). PS surfaces of a 24-well plate were used as controls (positive = hemolytic, negative = non-hemolytic). The different surface coatings to be analyzed and the negative control were each washed once with 1 mL of a 0.9% (w/v) NaCl solution (B. Braun Melsungen, Germany) and shaken on a plate shaker for 1 min at 300 rpm at 37°C. The washing solution was discarded, and the samples were equilibrated in 2,940 µL of fresh NaCl solution for 30 min at 37°C. The positive control was washed and equilibrated under the same conditions, using demineralised water instead of NaCl solution. The LH blood was diluted 4:5 in 0.9% NaCl solution. 60 μL of diluted LH blood was added to each equilibrated sample and control and incubated at 200 rpm and 37°C for 1 h. At the end of the incubation, each sample was treated at 400 rpm. From each sample and control, 950 µL of supernatant was transferred to a 1.5 mL reaction tube. The supernatants were centrifuged at 700 *g* for 10 min at room temperature (RT) (Centrifuge 5424 R with rotor FA-45–24-11, Eppendorf, Germany). Subsequently, 200 µL of the centrifuged supernatant was transferred to a 96-well plate (PS), and the absorbance was measured at 542 nm (CLARIOstar Plus, BMG LABTECH, Germany). The results were presented as hemolysis ratio (*HR*) in percent according to Eq. (2), where *A* is the absorbance of the sample, *C*_*neg*_ is the absorbance of the negative control and *C*_*pos*_ is the absorbance of the positive control. Samples and controls were analyzed in triplicate.

$$HR=\frac{A-C_{neg}}{C_{pos}-C_{neg}}\times 100\%$$ (2)

### 2.13 Human leucocyte activation assay

To investigate leukocyte activation of blood in contact with modified surfaces on coverslips, two samples of each modification were placed in a 6-well plate (one sample per well, PS). A PS surface was used as a negative control (duplicate). Samples and controls were disinfected once each with 3 mL of 70% (v/v) EtOH solution (Carl Roth, Germany), then washed once with 3 mL of 0.9% (w/v) NaCl solution (B. Braun Melsungen, Germany), and shaken on a plate shaker for 1 min at 300 rpm at 37°C. Venous blood was collected as described. To the samples and controls, 1.5 mL of LH blood was added and incubated at 37°C for 1 h at 200 rpm. As a positive control, 100 ng/mL lipopolysaccharide (LPS) from *E. coli* (Sigma Aldrich, Germany) was used. From each sample vial, 950 µL were transferred to a 1.5 mL reaction vial and centrifuged at 1,400 × *g* for 15 min at room temperature (RT). Meanwhile, the reagents for the polymorphonuclear (PMN) elastase assay (Demeditec Diagnostics, Germany) were prepared according to the manufacturer’s protocol. Briefly, 10 µL supernatant of the samples and the positive and negative controls were diluted 1:100 with calibrator/sample diluent (C/SD). 100 μL of controls and samples were added to the wells of a 96-well plate coated with polyclonal antibodies against PMN elastase. The samples were incubated for 1 h at 900 rpm and RT. The wells were then washed four times with 300 µL of 1x wash buffer before 150 µL of enzyme conjugate was added. After incubation under the same conditions, the wells were washed as before and 200 µL substrate solution was added. The plate was incubated for 20 min at RT with light excluded. 50 μL of stop solution was added, and absorbance was measured at 450 nm (CLARIOstar Plus, BMG LABTECH, Germany). Results were presented as leukocyte activation (*LA*) ratio in percent according to Eq. (3), where *A* is the absorbance of the sample, *C*_*neg*_ is the absorbance of the negative control, and *C*_*pos*_ is the absorbance of the positive control.

$$LA=\frac{A-C_{neg}}{C_{pos}-C_{neg}}\times 100\%$$ (3)

### 2.14 Statistical analysis of cytotoxicity and hemocompatibility assays

The values are reported as averages together with the standard deviation (the number of replicates is given in the results section). Statistical significance was determined using independent sample t-tests. The Bonferroni method was used to adjust for multiple comparisons. (Noble, 2009).

### 2.15 Dynamic *E. coli* attachment assay

Cell cultivation of *Escherichia coli* K12 (*E. coli,* DSM 498) was conducted according to previously reported protocols for *Cobetia marina* bacteria. (Schwarze et al., 2020). Though, the cultivation conditions were adapted for *E. coli*, so that Müller Hinton medium and an incubation temperature of 37°C were used. The attachment assay was conducted in M9 minimal medium with 1% glycerol on an orbital shaker at 60 rpm for 45 min with 7.5 ⋅ 10^6^ cells/mL according to previously established protocols. (Schönemann et al., 2021; Gnanasampanthan et al., 2022). ×80fields of view were recorded of adherent and with propidium iodide stained bacteria on coated microscopy slides using fluorescence microscopy (Texas Red filter set, Nikon Ti-E, ×10 objective Nikon CFI Plan Fluor DLL NA 0.3, Nikon Japan). The relative settlement density was determined by counting adherent bacterial cells. Outliers were removed when outside the 1.5 Interquartile range, and at least 60 fields of view per sample and three samples per coating were used to determine averages and standard deviations. Observed differences were tested for statistical significance with an ANOVA combined with a *post hoc* Tukey test at a significance level of α = 0.05.

### 2.16 Scanning electron microscopy

A scanning electron microscope (SEM, Quanta 3D FEG, FEI Company, Hillsboro, OR, United States) was used to image (20 kV, 3.0 spot size) the attachment of cellular components of human whole blood, the adhesion behavior of human thrombocytes and bacterial cells of an *E. coli* strain to the different surface coatings. Therefore, cultivated surfaces with whole blood or thrombocytes were fixed with 2.5% glutaraldehyde (50% in H_2_O, Sigma Aldrich, Germany) in DPBS (GIBCO, Life Technologies, United Kingdom) for 30 min followed by a triplicate wash with DPBS for 5 min. A secondary fixation was performed with 1% osmium tetraoxide (OsO_4,_ ≥ 99, 95%, Carl Roth, Germany) in DPBS for 15 min, and samples were rinsed again as described above. Dehydration was performed in a series of increasing ethanol concentrations from 30, over 50, 70, 96 to finally three-fold 100% leaving the samples 5 min in each solution. The dehydrated samples were air-dried in advance of sputtering. Adherent *E. coli* cells were air-dried. The samples were coated with a ∼11 nm thick layer of gold using sputter coating deposition (Cressington Scientific Instruments Ltd, Watford, United Kingdom) before imaging to prevent charging effects during the measurements.

## 3 Results

**FIGURE 1:**
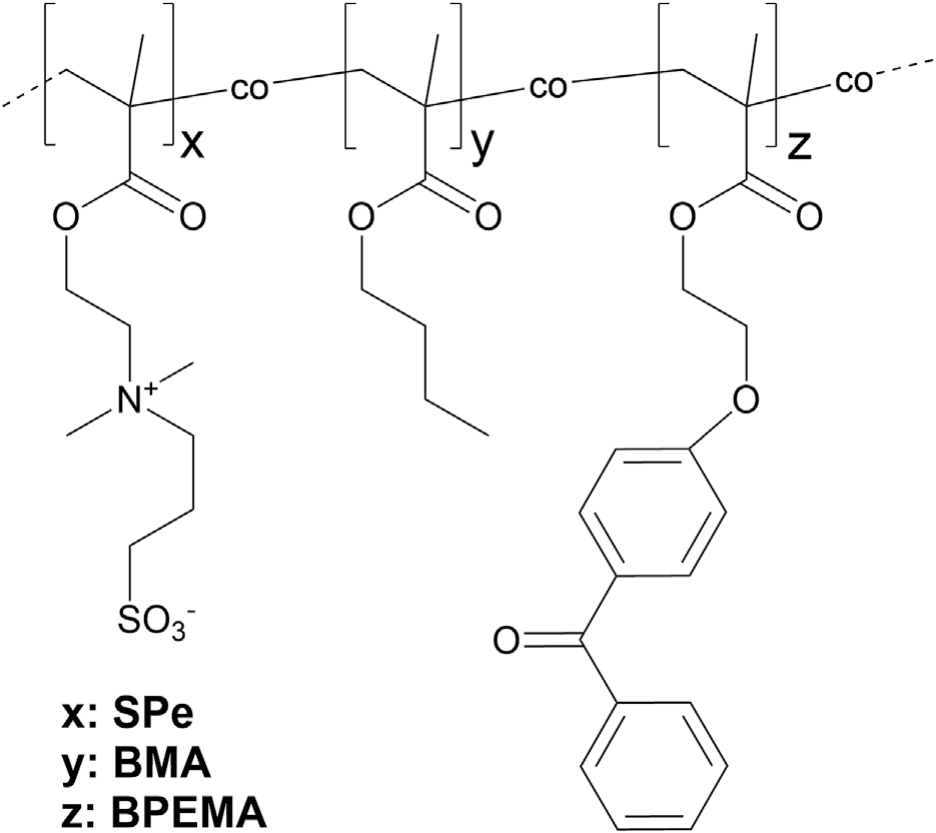
Schematic chemical structure of copolymers made of hydrophobic pBMA, zwitterionic pSPe, and crosslinker BPEMA in variable proportions according to Table 1.

Synthesis of pBMA, pSPe, and amphiphilic sulfobetaines was carried out according to previously established protocols via free radical co-polymerization. (Schardt et al., 2022). Within the amphiphilic polymers, the contents of BMA were varied from 10 to 50 mol%, and of SPe from 90 to 50 mol%, respectively. All formulations contain 1 mol% of BPEMA as photo-crosslinker (s. Figure 1). 1 wt% of polymer was dissolved in trifluoroethanol (TFE), spin coated onto amino propyl trimethoxy silane (APTMS) functionalized Si-surfaces, and cured by UV irradiation. (Prucker et al., 2018; Koc et al., 2019). The thickness of the obtained polymer coatings was determined by spectroscopic ellipsometry, and the wettability was characterized by contact angle goniometry (water contact angle WCA and captive bubble contact angle CBCA) (Table 1).

**TABLE 1:** Sample codes, composition, contact angles in air and water, and thickness of the polymer coatings. Reported CBCA values were determined immediately after immersion.

Sample	SPe content x [mol%]^a^	BMA content y [mol%]^a^	BPEMA content z [mol%]^a^	WCA [°]	CBCA [°]	Thickness [nm]
pBMA	-	99	1 ± 0.3	78 ± 6	81 ± 2	97 ± 14
cop-SPe-5-BMA-5	53	46	1 ± 0.3	60 ± 3	20 ± 1	108 ± 10
cop-SPe-7-BMA-3	72	27	1 ± 0.3	59 ± 3	19 ± 2	104 ± 6
cop-SPe-9-BMA-1	88	11	1 ± 0.3	36 ± 3	15 ± 2	120 ± 11
pSPe	99	-	1 ± 0.3	17 ± 3	13 ± 2	128 ± 10

^a^ Data from Schardt *et al*. (Schardt et al., 2022), values are only reliable ±10 rel.% for SPe/BMA, content and ±50 rel.% for BPEMA, content.

**FIGURE 2:**
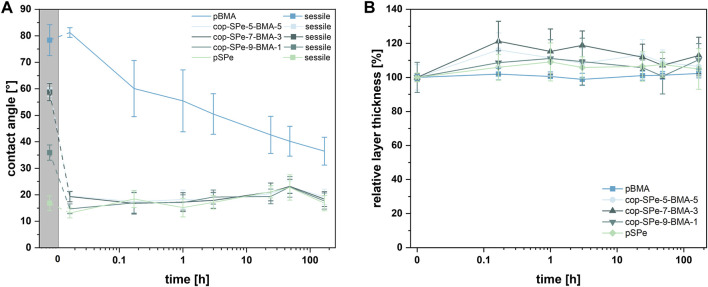
**(A)** Sessile (squares, grey background) and captive bubble contact angles, and **(B)** the relative thickness of each coating is provided by the measured thickness of the polymer, when compared to its initial thickness prior to the immersion (0 h). This normalized thickness is used to assess changes in the coating’s thickness over time in relation to its original dimensions. Each data point represents the average of three measurements on three different samples on pBMA, cop-SPe-5-BMA-5, cop-SPe-7-BMA-3, cop-SPe-9-BMA-1 and pSPe with corresponding SD (n = 9) after different incubation periods in 0.5 x PBS solution.

Hydrophobic pBMA exhibits the highest WCA with 78° ± 6°. With increasing zwitterion content, the static WCA decreases. While the high WCA of pBMA remains similar directly after immersion, the CBCA of the SPe-containing coatings drops immediately down to 13°–20°. The differences in the contact angle of the amphiphilic polysulfobetaines as a function of BMA content can also be observed directly after immersion in water with cop-SPe-5-BMA-5 being the highest (20°) and pSPe being the lowest (13°), albeit some of the differences are within the error bars. At the same time, with an increase of the hydrophilic SPe content, the polar proportion of the surface energy also increases, while the dispersive proportion remains almost unchanged (cf. Supporting information, Supplementary Figure S1). All polymer coatings were incubated in 0.5 x PBS for up to 7 days with regular control of CBCA and layer thickness. Figure 2A shows that after the initial rearrangement, the SPe-containing polymers reveal nearly unchanged CBCAs between 13° and 25° over 7 days. For the hydrophobic pBMA, a steady decline of the CBCA down to 36° ± 5° is observed. To determine if the relative thickness changes over the immersion time of 7 days, thicknesses were also assessed at each time point and normalized to the initial values which were between 100 and 130 nm (Table 1). All coatings retain a thickness close to 100% (Figure 2B) with pBMA retaining constant layer thickness over the entire incubation and some of the zwitterionic coatings showing slightly higher thicknesses, albeit most of the changes are within the error bars.

**FIGURE 3:**
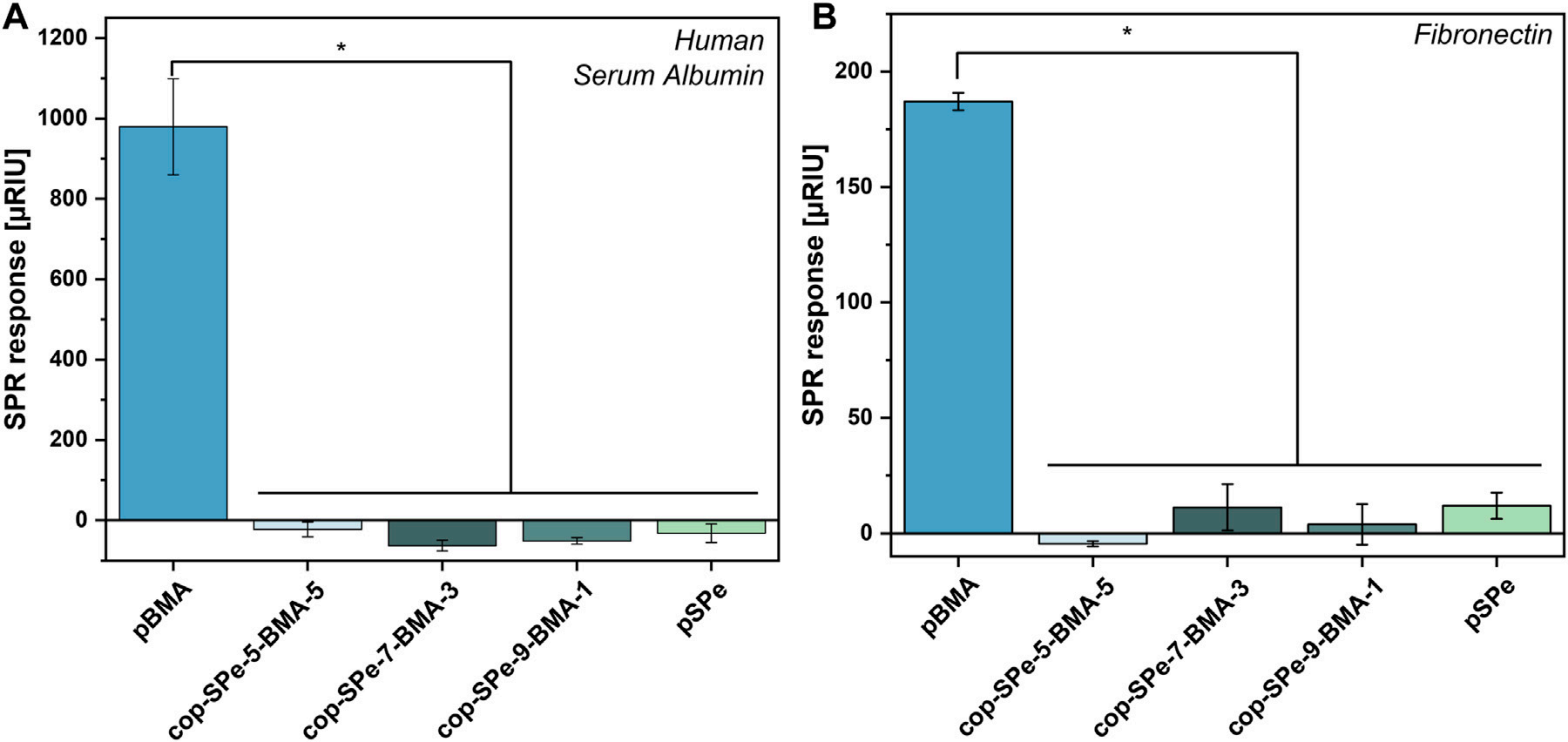
Protein resistance measurement by SPR of **(A)** 1 mg/mL human serum albumin and of **(B)** 0.05 mg/mL fibronectin of pBMA, cop-SPe-5-BMA-5, cop-SPe-7-BMA-3, cop-SPe-9-BMA-1, and pSPe. Values describe the SPR signal change after completion of protein injection averaged over three measurements with corresponding SEM, *p < 0.05.

Figure 3 shows the NSA of protein measurements with two different proteins. Compared to the negative control pBMA, all SPe-containing polymers exhibit an extremely low amount of NSA, for both HSA and Fn (*p* < 0.05). Within the amphiphilic series, all surfaces are highly resistant against the adsorption of both proteins and there are no significant differences in their ability to resist protein adsorption.

**FIGURE 4:**
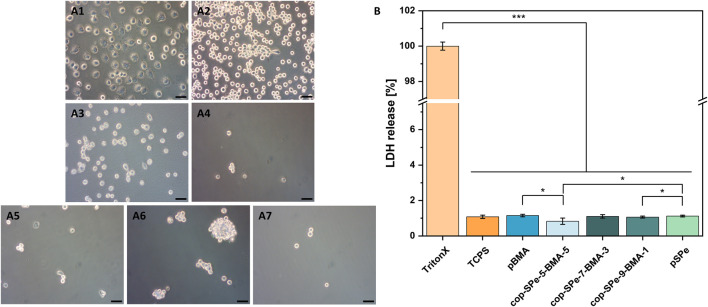
**(A)** Bright field live cell (BFLC) images show L929 mouse fibroblasts after cultivation for about 24 h on **(A1)**: TCPS, **(A2)**: PS, **(A3)**: pBMA, **(A4)**: cop-SPe-5-BMA-5, **(A5)**: cop-SPe-7-BMA-3, **(A6)**: cop-SPe-9-BMA-1, and **(A7)**: pSPe. The scale bar corresponds to 50 µm. **(B)** Quantitative analysis of the cell membrane integrity assay by LDH release.

In accordance with DIN EN ISO 10993–5 (*In vitro* cytotoxicity analysis), the different coatings were brought into direct contact with the L929 mouse fibroblast cell line. Figure 4 shows the bright field live cell (BFLC) imaging microscopy after a cultivation period of approx. 24 h on the respective surfaces of pBMA, cop-SPe-5-BMA-5, cop-SPe-7-BMA-3, cop-SPe-9-BMA-1, pSPe, and the controls tissue culture polystyrene (TCPS) and polystyrene (PS).

Typical of TCPS (positive control for excellent cell adhesion), more than 95% of the initially seeded L929 mouse fibroblasts show strong adhesion after more than 20 h of standard cultivation (Figure 4A1). The cell bodies are also typically spread out on the surface and show multiple filopodia for palpation of the surface and contact with neighboring cells. In contrast, the L929 mouse fibroblasts on the PS surface, which is intended for cell proliferation in suspension culture, are only weakly adherent in the time window considered. With a few exceptions, they are almost exclusively round and not spread (Figure 4A2). On the pBMA surface, these cells show stronger adherence and lower nuclear-plasma ratio compared to PS, i.e., the L929 fibroblasts are slightly more spread (Figure 4A3). On the pSPe surface and the amphiphilic sulfobetaine surfaces, the L929 cells can no longer adhere. The BFLC images in Figure 4A4–A7 show only very few, round L929 cells that did not adhere to the surface coating. Figure 4A6 shows a surface section of coating cop-SPe-9-BMA-1, on which a few very mobile L929 cell aggregates can be seen. The sections shown in Figure 4A4–A7 are the only ones on which cells can be seen at all in relation to the total surfaces examined under the microscope (for details cf. Supporting information, Supplementary Table S2). A quantitative cell membrane integrity assay was used to prove that the prevention of cell adherence is not a cytotoxic effect. Figure 4B shows no cytotoxic effect during direct incubation of L929 cells compared to the TCPS standard surface.

**FIGURE 5:**
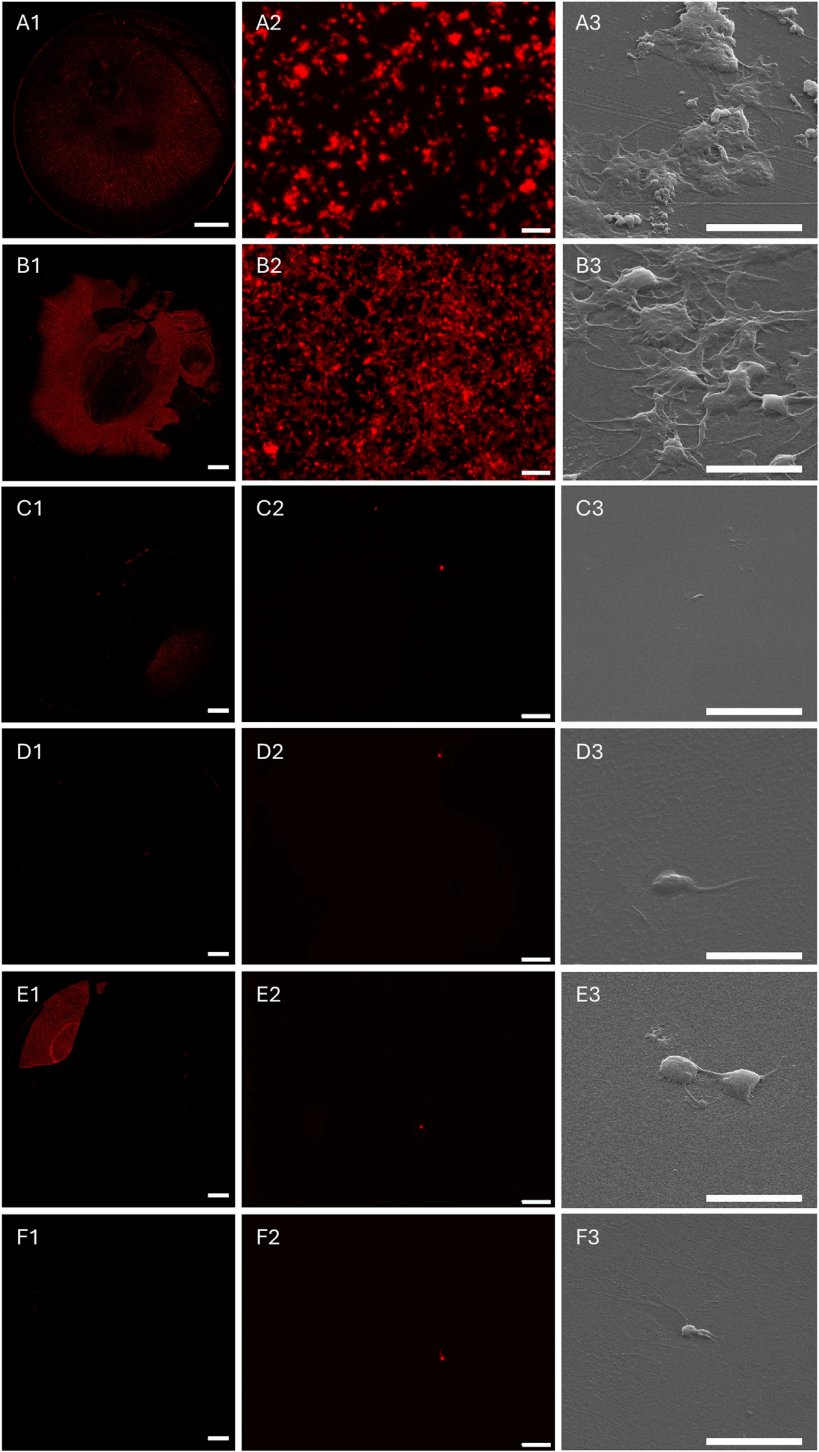
Fluorescence microscopy (FM) images of the entire sample surface (left column) and a corresponding characteristic sample surface section after incubation with human blood platelets whose F-actin was labeled with phalloidin-546 (mid column), as well as a characteristic sample surface section using SEM (right column) on **(A1–A3)**: TCPS, **(B1–B3)**: pBMA, **(C1–C3)**: cop-SPe-5-BMA-5, **(D1–D3)**:, cop-SPe-7-BMA-3, **(E1–E3)**: cop-SPe-9-BMA-1, and **(F1–F3)**: pSPe. FM images: **(A1–F1)**: The scale bar corresponds to 2 mm; **(A2–F2)**: The scale bar corresponds to 20 μm; **(A3–F3)**: SEM images: The scale bar corresponds to 5 µm.

**FIGURE 6:**
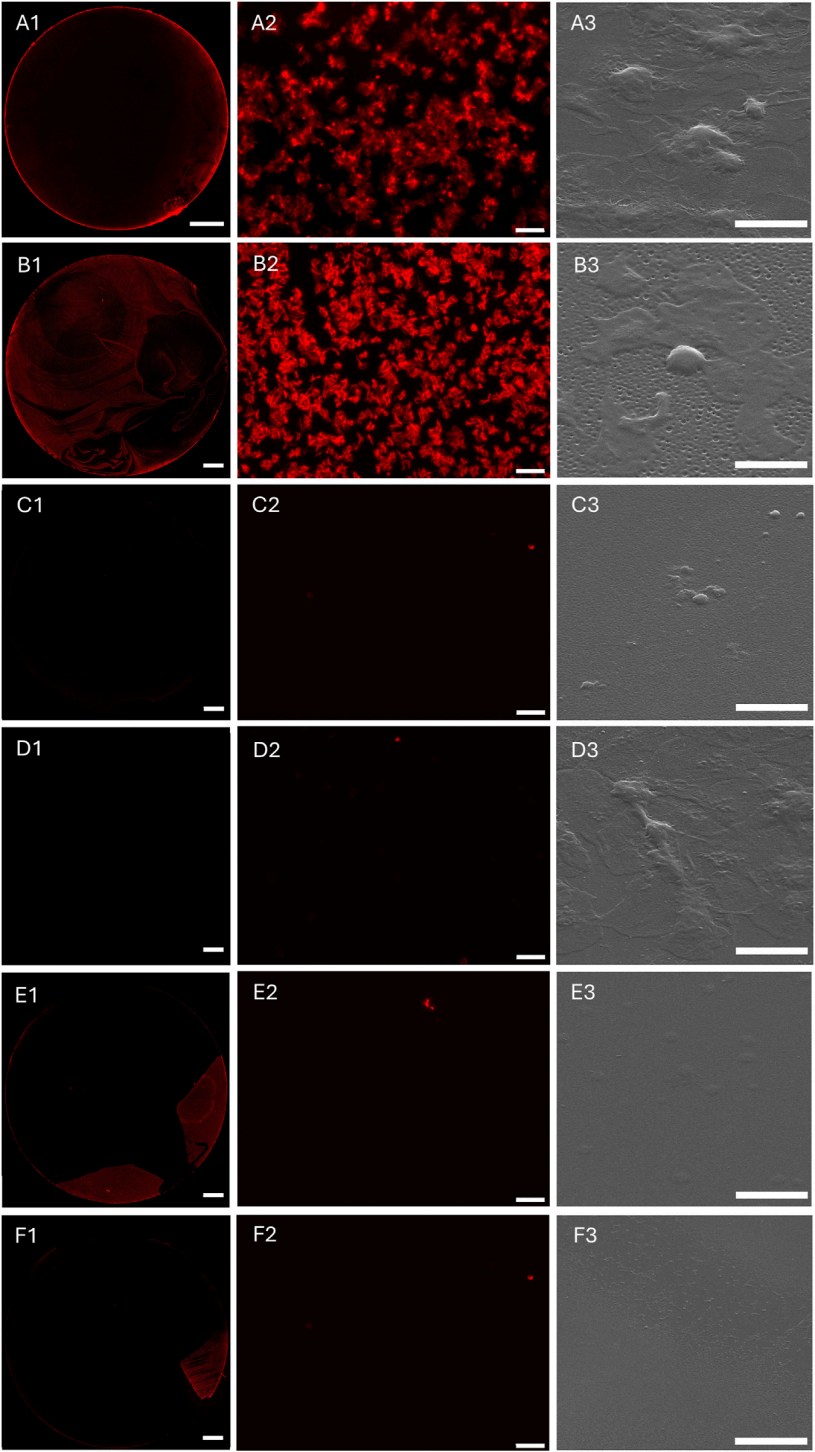
Fluorescence microscopy (FM) images of the entire sample surface (left column) and a corresponding characteristic sample surface section after incubation with human whole LH blood (mid column). F-actin adherent cells were labeled with phalloidin-546. A characteristic sample surface section was analyzed by SEM (right column) for **(A1–A3)**: TCPS, **(B1–B3)**: pBMA, **(C1–C3)**: pSPe, **(D1–D3)**: cop-SPe-5-BMA-5, **(E1–E3)**: cop-SPe-7-BMA-3 and **(F1–F3)**: cop-SPe-9-BMA-1. FM images: **(A1–F1)**: The scale bar corresponds to 2 mm; **(A2–F2)**: The scale bar corresponds to 20 μm; SEM images: **(A3–F3)**: The scale bar corresponds to 5 µm.

In further experiments, the different polymer coatings were examined regarding their interaction with human platelets (platelet adhesion assay, Figure 5) and human whole blood (whole human LH blood adhesion assay, Figure 6) in accordance with DIN EN ISO 10993–4 (testing for interaction of biomaterials or medical devices with blood). For this purpose, the sample surfaces were incubated with platelet-enriched plasma (PRP) or LH whole blood under controlled dynamic conditions for 1 h. In Figures 5, 6, the FM images of the entire incubation surface of the control TCPS (A1) as well as the different coatings pBMA (B1), cop-SPe-5-BMA-5 (C1), cop-SPe-7-BMA-3 (D1), cop-SPe-9-BMA-1 (E1), and pSPe (F1) are shown under A1-F1. The surfaces TCPS as a positive control for cell adhesion and pBMA show strong platelet adhesion, spreading, and also partial aggregation in both tests. The observation of the respective SEM images shows no adhesion of other blood cells, but an additional increased adsorption of soluble blood components on the TCPS and pBMA sample surfaces incubated with LH whole blood. On the pSPe-modified surface (Figure 5F1–F3, Figure 6F1–F3), both the adhesion and the aggregate formation of the platelets are significantly reduced. As an example, on a pSPe surface incubated with LH whole blood, a coating defect with stronger platelet adhesion can be seen in the lower right image of the FM image (Figure 6F1). Apart from very few coating defects, the FM and SEM images for all surfaces with amphiphilic sulfobetaine coatings from cop-SPe-5-BMA-5 (Figure 5C1–C3, Figure 6C1–C3), cop-SPe-7-BMA-3 (Figure 5, Figure 6D1–D3) to cop-SPe-9-BMA-1 (Figure 5, Figure 6E1–E3) show that the extent of adhesion of human platelets decreases successively and aggregation is no longer visible. In addition, unlike on TCPS and pBMA, the high-resolution SEM images do not show adhesion of other cells or adsorption of other blood components.

**FIGURE 7:**
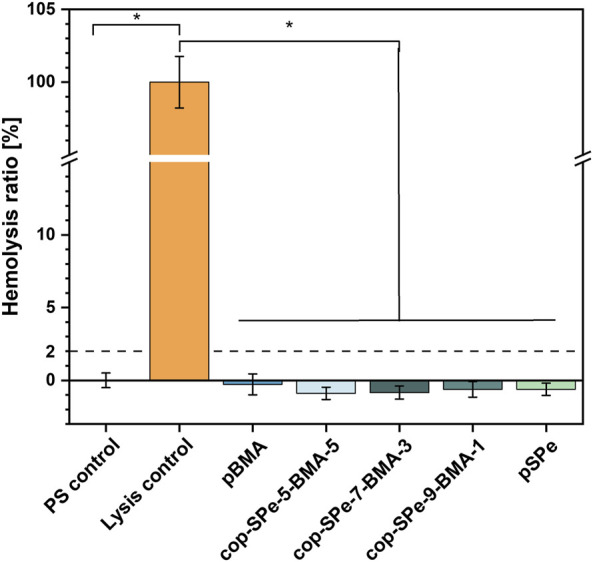
Investigation of hemolysis of agitated human erythrocytes on the different surface modifications after 1 h incubation of diluted LH blood at 37°C. The hemolysis of the empty wells (PS) was set to 0 (blood incubated in NaCl solution), while the lysis control (blood incubated in dist. H_2_O) corresponds to 100%. Dashed line represents the 2% threshold, indicating that the biomaterial is considered non-erythrotoxic. Measurements were carried out in triplicates, *p < 0.05.

The dynamic hemolysis assay was used to investigate whether freshly isolated human erythrocytes undergo cell membrane destabilization in contact with the different surface coatings, to release hemoglobin into the supernatant. Like the control surface PS, all the coatings examined showed no erythrotoxic effect in the sense of membrane destabilization and resulting hemolysis (Figure 7). For comparison, incubation with demineralised water and the resulting osmotic pressure leading to bursting of the RBCs provided the control value with complete hemolysis.

**FIGURE 8:**
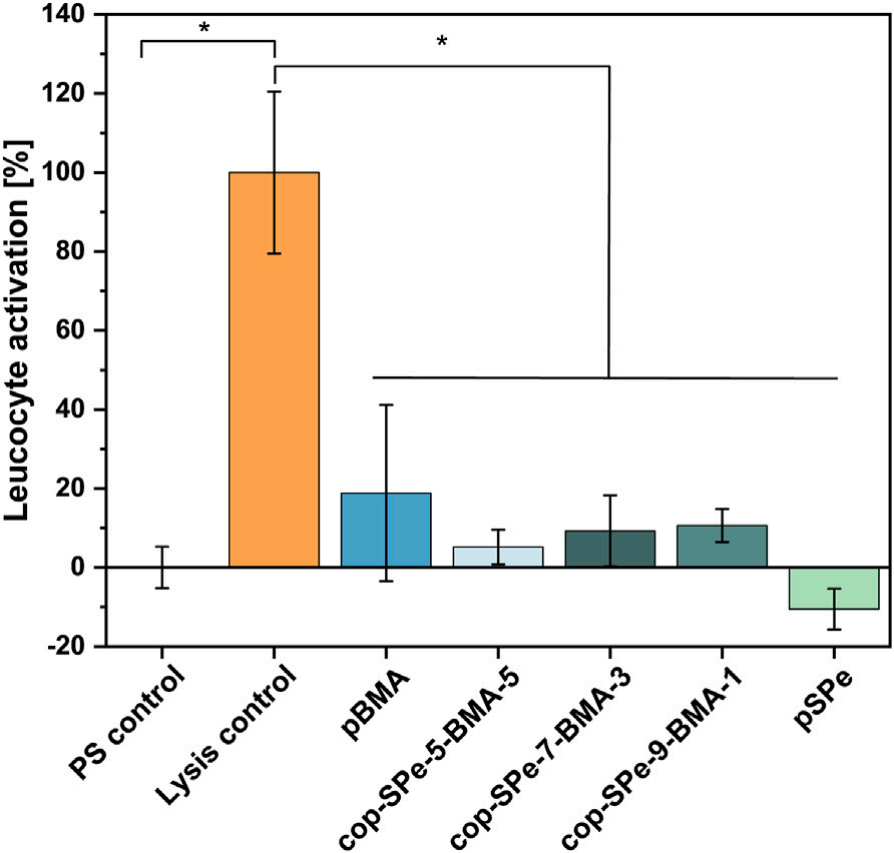
Leucocyte activation during a 1 h blood/biomaterial interaction was analyzed using PMN elastase ELISA assay. The baseline PMN elastase expression in non-activated blood in an empty PS well was set to 100%. The leucocyte activation of the empty wells (PS) was set to 0, while the exemplary induction of leucocyte activation was achieved by the addition of bacterial lipopolysaccharides and corresponds to 100% (Lysis control). Measurements were carried out in duplicates. All coatings used showed no significant increase in leukocyte activity, *p < 0.05.

The extent of PMN elastase expression is an indicator of leukocyte activation, as inflammatory stimulation of neutrophils—the most abundant leukocytes—leads to a rapid release of large amounts of this proteolytic enzyme. Therefore, the release of PMN elastase after 1 h exposure of whole blood to the surface coatings pBMA, pSPe, cop-SPe-5-BMA-5, cop-SPe-7-BMA-3, and cop-SPe-9-BMA-1 was analyzed (Figure 8). None of the coatings showed a significant increase in leukocyte activity.

**FIGURE 9:**
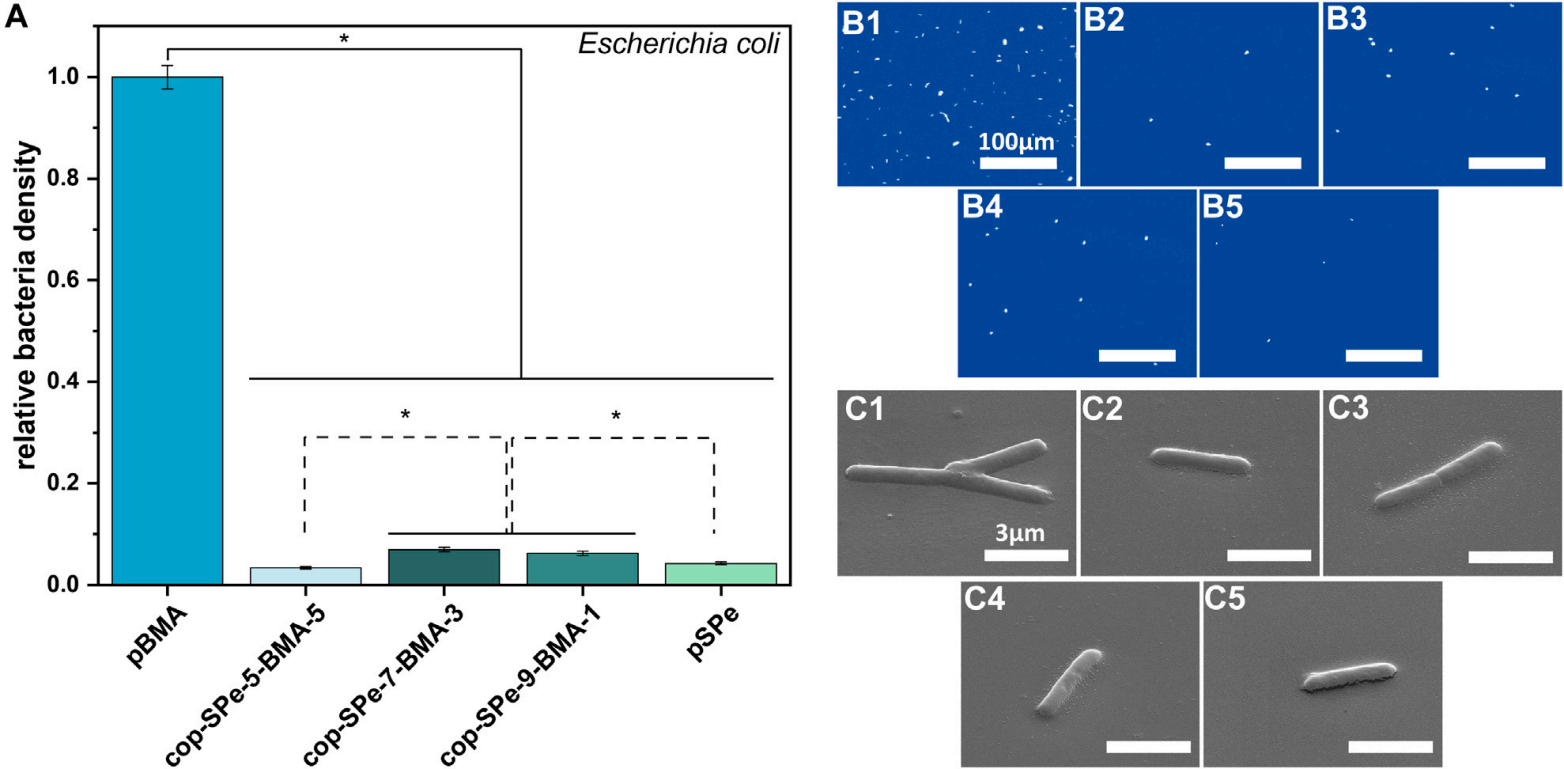
**(A)** Relative density of *Escherichia coli* on pBMA, cop-SPe-5-BMA-5, cop-SPe-7-BMA-3, cop-SPe-9-BMA-1 and pSPe normalized to the hydrophobic control pBMA. Dynamic accumulation was conducted with 7.5.10^6^ cells/mL, at 60 rpm on an orbital shaker for 45 min. Values represent the average of three measurements with at least 60 fields of view with corresponding SEM. Statistically significant differences are marked with an asterisk *p < 0.05. Statistically significant differences among the zwitterionic samples are marked with a dotted line. **(B1-5)** Exemplary microscopy images (scale bar corresponds to 100 µm) and **(C1-5)** SEM images captured with 45°-tilted stage (scale bar corresponds to 3 µm) of the *Escherichia coli* attachment assay on (1) pBMA, (2) cop-SPe-5-BMA-5, (3) cop-SPe-7-BMA-3, (4) cop-SPe-9-BMA-1, and (5) pSPe.

Besides protein resistance and hemocompatibility, also bacterial resistance is a key feature for implantable biomedical devices. Therefore, the resistance of the homo- and copolymer coatings against the attachment of the bacterium *E. coli* under dynamic conditions was tested (Figure 9). The SPe-containing polymers accumulate only 3%–7% of the *E. coli* density on pBMA. The reduction was in all cases statistically significant (*p* < 0.05). The lowest accumulations were found on cop-SPe-5-BMA-5 (3.3% ± 0.2%) and pSPe (4.3% ± 0.3%), followed by cop-SPe-9-BMA-1 (6.2% ± 0.4%) and cop-SPe-7-BMA-3 (7.0% ± 0.4%). When pBMA was included in the statistical analysis, no significant difference was seen between the different zwitterionic polymers (Figure 9). However, when restricting the statistical analysis only to the zwitterionic coatings, cop-SPe-5-BMA-5, and pSPe showed a statistically lower attachment compared to cop-SPe-9-BMA-1 and cop-SPe-7-BMA-3 (Figure 9 dotted line, *p* < 0.05). Single adherent bacteria are uniformly distributed on the different surface coatings (Figure 9B1–B5). The SEM images illustrate the integrity of the bacteria showing undamaged bacterial membranes (Figure 9C1–C5).

## 4 Discussion

Copolymerization of hydrophobic BMA with hydrophilic SPe yielded polymer coatings with variable amphiphilic character, which show high stability under physiological conditions. The amphiphilic character became obvious in the WCA measurements, as higher BMA contents enhanced the hydrophobicity of the coatings in the dry state. However, upon immersion in aqueous media (PBS), all the zwitterion-containing coatings showed a fast reorientation with strongly decreased CBCAs independent of their hydrophobic content. The low CBCAs indicate that the zwitterionic moieties accumulate at the interface while the hydrophobic moieties reorient inside the film. This is consistent with previous reports in which the reorientation behavior was studied in saltwater. (Schardt et al., 2022). A much slower and much less pronounced CBCA decrease was observed for pure pBMA reaching 36° ± 5° after 168 h. CBCA measurements in MilliQ water show a constant contact angle of 80° after 1 h immersion (Koschitzki et al., 2021), which is similar to the CBCAs of pBMA in PBS after 1 h. Literature reports that 7 days immersion of pBMA coatings in saltwater led to a reduction of the CBCA to approx. 55°. (Schardt et al., 2022). The even more pronounced reduction of the contact angle of pBMA in PBS seems therefore not solely attributed to the ion strength alone. We speculate that in particular, the interactions with the phosphates could be responsible for the observed decrease.

The resistance of the amphiphilic coatings against non-specific protein adsorption of proteins was tested against HSA and Fn. While HSA is the most abundant protein in blood, (Evans, 2002), Fn is known to be involved in cell adhesion, migration, and differentiation processes. (Grinnell and Feld, 1981; Iuliano et al., 1993; García et al., 1997). Pure pSPe and all amphiphilic coatings with up to 50% BMA content resist the NSA of HSA and Fn effectively. For both proteins, the NSA on pBMA is at least 16 times higher than on the zwitterionic samples. Even polymers with elevated contents of up to 50% BMA effectively suppressed the NSA of proteins (Figure 3). All coatings with low nonspecific protein adsorption showed low underwater contact angles with CBCAs ≤20°, which were observed even with high contents of the hydrophobic BMA. Previous studies have investigated the influence of increasing hydrophobic content in poly (oxanorbornene) by changing the length of peripheral alkyl chains from methyl over ethyl, butyl, hexyl to octyl. While ethyl and butyl groups do neither affect the wettability nor the NSA of proteins, an increase of the alkyl chain length from hexyl to octyl increased the WCA and CBCA substantially and increased the NSA of fibrinogen (Fg). (Paschke et al., 2022). Literature also reported that copolymers consisting of PC and BMA showed increasing resistance with increasing zwitterionic content with the highest resistance achieved by the highest zwitterionic content of 30%. (Ishihara et al., 1992). Lin and co-workers copolymerized acrylate-based CB with BMA by free radical polymerization whereby the zwitterionic contents range from 17% to 82%. Copolymers with CB compositions higher than 31% detached from the surface. Simultaneously, copolymers with 31% CB content had the highest hydrophilicity and the lowest Fg adsorption. (Lin et al., 2019). On the one hand, the detachment of the coatings with higher zwitterionic content can lead to a higher hydrophobicity. On the other hand, intra- and intermolecular charge compensation of the different zwitterionic groups may render the overall polymer more hydrophobic. (Walker et al., 2019). The WCA of our samples increases with increasing hydrophobic content, however, this phenomenon is less pronounced under water (CBCA). No detachment was observed, even if the zwitterionic content was above 50%. This is probably due to the use of the adhesion promoting layer (APTMS) under, and of crosslinkers within the copolymers.

Body fluids and especially the acellular part of the blood, the plasma, have an extremely complex composition. If the water-soluble Fg, the main component of plasmatic coagulation, is removed from the plasma, then serum remains after centrifugation. Most invasive medical implants come into contact with blood, and thus, with a higher concentration of proteins. These can adsorb to biomaterial surfaces to varying degrees non-specifically. Subsequently, either cells circulating in the blood (platelets, erythrocytes, leucocytes, etc.) or, depending on the implantation site, localized connective tissue cells can adhere to the biomaterial surface contaminated with proteins, among other things. (Curtis and Forrester, 1984; Steele et al., 1992; Hall-Stoodley et al., 2004; Zander and Becker, 2018). In this context, the cultivation experiments with L929 mouse fibroblasts seeded in RPMI medium with a 10% FCS addition on the surface coatings with 90–50 mol% SPe and pure pSPe show no tendency to adhere within 24 h despite the high serum protein content. This result is in line with the low NSA of HSA, Fn (Figure 3) and Fg (Schardt et al., 2022) on these coatings as shown by the protein resistance measurements by SPR. As Fb and Fn are directly involved in many cell adhesion mechanisms, the NSA of these proteins is highly relevant for the cell adhesion process on the coatings. (Lin et al., 2019). Hydrophilic SB-based coatings without fibroblast adhesion have been previously reported in the literature (Kasák et al., 2011; Stach et al., 2011), which is consistent with the high resistance of the pure pSPe coatings in our experiments. Also, amphiphilic coatings containing PC (Ishihara et al., 1999) or CB (Lin et al., 2019) as zwitterionic moiety have extensively been studied, successfully preventing fibroblast adhesion. Yet reports on the hemocompatibility and leukocyte activation of amphiphilic sulfobetaines with systematically changing amphiphilicity are still lacking in the literature. Even though there are significantly fewer L929 cells on the initially hydrophobic pBMA surfaces than on TCPS and PS, some cells show the formation of pseudopodia, spreading and thus adhesion to the surface. The mobility of the few fibroblasts is also comparable to that on PS, an observation that could be made during microscopy. Low NSA of proteins and decreased mobility of L929 can be seen on pBMA. Serum albumin and Fn are both components of FCS, increased NSA of these proteins may be associated with increased binding of L929 fibroblasts. (Curtis and Forrester, 1984; van Wachem et al., 1987; Steele et al., 1992). In this context, the described correlation in the literature is supported by the simultaneous occurrence of high NSA of these proteins (Figure 3) and the reduced mobility of L929 on pBMA.

Platelets (also called thrombocytes) have a key function in the sum of physiological processes that cause bleeding to stop by clot formation. Specifically, they are at the beginning of a series of cascades called primary hemostasis. In the course of a vascular injury, in which, for example, the intima, i.e., the intraluminal endothelial cell layer, is damaged, proteins of the basal lamina lying below the endothelial cells, such as collagen IV and especially Fn, are exposed. This leads to rapid platelet adhesion, activation, and aggregation. (Berndt et al., 2014). Any insertion of a biomaterial in the form of an invasive implant, i.e., a foreign body, is first of all a potentially disruptive process to normal physiology. It is well known that the insertion of, for example, a temporary central venous or peripheral arterial catheter leads to the formation of blood clots on the inner wall of the catheter. It is also well known that platelets are responsible for this in the context of primary hemostasis, which spontaneously adhere to the foreign surface mediated by NSA of mainly Fg (Struczyńska et al., 2023), but also Fn(Lin et al., 2019) and other glycoproteins. (Ward, 2008). Consequently, it is not surprising that platelets on the pBMA surface with low NSA resistance to Fg (Schardt et al., 2022), HSA and Fn show also pronounced adhesion and aggregation. In comparison, it is expected that when surfaces are coated with cop-SPe-5-BMA-5, cop-SPe-7-BMA-3, and cop-SPe-9-BMA-1, platelet adhesion decreases with increasing proportion of SPe. Indeed, on the intact pSPe surface, the number of adherent and aggregated platelets is significantly lower compared to the TCPS control and pBMA surface. Interestingly, however, it is larger than on the cop-SPe-9-BMA-1 surface. This illustrates that the previous experiments on blood glycoprotein adsorption alone are not sufficient to test the hemocompatibility of biomaterial surfaces, but only the interaction of primary blood cells (here primary human platelets) moving across and scanning the biomaterial surface with their pseudopodia, minute amounts of NSA blood proteins can be detected and lead to this minor platelet adhesion and aggregation on pSPe. Considering that from cop-SPe-5-BMA-5 via cop-SPe-7-BMA-3 to cop-SPe-9-BMA-1, the sulfobetaine 3-[N-2-(methacryloyloxy)ethyl-N,N-dimethyl]-ammonio propane-1-sulfonate (SPe) content increases but the amount of adhering platelets decreases, the particular importance of a specific molecular arrangement of the hydrophilic (SPe) and hydrophobic (BMA) portions of the coatings studied becomes more understandable under physiological conditions. The anti-platelet adhesion and activation properties of the poly (SPe-co-BMA) coincide with previous studies with poly (MPC-co-BMA) polymers. The hydrophilic MPC moiety seems to successfully suppress platelet adhesion and impart the non-thrombogenic properties of the coatings. (Ishihara et al., 1990a; Ishihara et al., 1992). A low platelet adhesion was also reported for poly (CBMA-co-BMA) with zwitterionic contents up to 30%. (Lee et al., 2021). In the context of platelet adhesion and all subsequent platelet reactions with foreign material surfaces, the amphiphilic sulfobetaine-modified surfaces also showed a very good effect against unspecific interaction with or adsorption of soluble blood components in the whole blood experiment, as well as platelet adhesion (Figures 5, 6).

Another aspect of blood coming into contact with biomaterial surfaces is the interaction of red blood cells (RBC), the oxygen-transporting erythrocytes that make up the largest proportion of cells in blood, with the foreign material surface. Here it is important to exclude indirect or direct erythrotoxic effects that may lead to a loss of RBC membrane integrity and thus to hemolysis. (Soundararajan et al., 2018; Pacharra et al., 2019; Podsiedlik et al., 2020). From this perspective, we have also investigated the surface coatings described here with regard to their erythrotoxicity, following the work of van Oeveren (van Oeveren, 2013) and Wang *et al.*. (Wang et al., 2013). According to numerous scientific papers, a biomaterial is considered non-erythrotoxic if the measured hemolysis rate is less than 2%. (He et al., 2023). This threshold is recommended in DIN EN ISO 10993–4 and ASTM F756-17. All surface coatings, including the amphiphilic ones with BMA contents up to 50%, investigated here are below this threshold and do not trigger any RBC membrane integrity loss and thus no hemolysis.

In addition to the platelets and the RBCs, neither of which has a cell nucleus, another cell nucleated, very heterogeneous cell population of the blood circulates through the human organism, the so-called white blood cells (WBCs) or leukocytes. These immunocompetent cells consist of granulocytes, lymphocytes, monocytes, mast cells, and dendritic cells. The segment-nucleated neutrophil granulocytes, casually referred to as neutrophils, account for the largest percentage of the total number of leukocytes (50%–70%). (Segal, 2005). As soon as the physiological integrity of the organism is disturbed, such as by invasive pathogens, intended (surgery) or unintended (accident) injuries, or by the introduction of a temporary or permanent medical object, neutrophils immediately move to that site, attracted by so-called pathogen-associated molecular patterns (PAMPs) or damage-associated molecular patterns (DAMPs). In the context of the relatively short activity period or life span of neutrophils, which is currently still very controversial in the literature and is put at 5–135 h, neutrophils are involved in the acute host-biomaterial interaction. (Zindel and Kubes, 2020; Macias and Keselowsky, 2022). In order to test this acute neutrophil reaction in interaction with the surface coatings under investigation here, a polymorphonuclear elastase (PMNE) assay was used. The enzyme PMNE is contained in the granules of neutrophils and released in the event of their activation by PAMPs or DAMPs. (Pacharra et al., 2019; 2020). The lipopolysaccharide (LPS) used as a control in the study of the interaction of neutrophils with the surface coatings pBMA, cop-SPe-5-BMA-5, cop-SPe-7-BMA-3, cop-SPe-9-BMA-1, and pSPe, is a component of the outer layer of the outer cell membrane of Gram-negative bacteria such as *E. coli*, and is a so-called PAMP. Thus, this elicits a strong PMNE response. Since none of the surface modifications showed as strong an activation as LPS or, in comparison, not even a significant response in the PMNE ELISA assay, this can be taken as an additional preliminary positive indication that especially the coatings with 90–50 mol% of SPe should be further investigated for use in finishing invasive medical devices to improve their hemo- and immunocompatibility.

It has been reported that SPe-containing polymers can not only prevent the adhesion of proteins, but also of bacteria. (Cheng et al., 2007; Li et al., 2008; Lalani and Liu, 2012; Schardt et al., 2022). The accumulated bacteria densities on all prepared zwitterion-containing coatings is <7% of the accumulation density on pBMA, and thus very low. Even an increase of the hydrophobic content of up to 50% creates still coatings with a high resistance to bacterial attachment and no formation of agglomerates can be seen. These findings show that the amphiphilic methacrylate copolymers containing hydrophilic sulfobetaine and hydrophobic butyl groups reduce bacterial attachment even at BMA contents of up to 50%. Our results concur well with previous studies in which copolymers composed of hydrophilic SB methacrylate with N-isopropyl acrylamide in 100:0, 70:30, 50:50, and 80:20 M ratios were shown to possess good protein resistance, low platelet adhesion, and resistance against the adhesion of human fibroblast and bacteria that increased with increasing zwitterionic content. (Chang et al., 2010). In agreement with our data, even small quantities of SB or other zwitterionic components induced already high hydrophilicity of the resulting coatings. This could in part be the reason for the high resistance against the attachment of several cell types. (Ishihara et al., 1990a; Ishihara et al., 1992; Chang et al., 2010).

Our study clearly shows that a certain degree of amphiphilicity seems to be advantageous for antiadhesive biomedical coatings. Further investigations with even smaller quantities than 50% of SB-type monomers could complement these results shown here and may result in even improved coatings, or provide information on whether there is a minimum of SB required for antiadhesive, antithrombogenic, and immunocompatible coatings. Future work might also investigate the interactions with the complement system, in particular with the factors C3 and C5, before eventually conducting preclinical *in vivo* trials to surpass commercially available coatings.

## 5 Summary and conclusion

Biomedical applications place high demands on biocompatible coatings regarding the prevention of the non-specific adsorption of proteins, the non-specific adhesion of human cells, and pathogenic microorganisms such as bacteria. Zwitterionic coatings are promising candidates as due to their hydrophilic properties and high hydration they show a remarkable resistance against various accumulation processes. (Ishihara et al., 1998; Ishihara, 2019; Lin et al., 2019). Here we were able to copolymerize hydrophilic SPe and hydrophobic BMA in different ratios to generate amphiphilic, easily applicable, photocrosslinkable polymer coatings. The resulting homo- and copolymer coatings are very stable in PBS solution and in addition, comprise a high content of SPe. The pure hydrophilic pSPe and also the amphiphilic coatings with up to 50% BMA are consequently very hydrophilic and provide high resistance against the NSA of proteins. The amphiphilic SPe/BMA copolymer coatings inhibit fibroblast adhesion without concomitant cytotoxicity and outperform the pure SPe coatings. The coatings also prevent the adhesion, activation, and thus aggregation of human platelets, not only when the platelets have been previously isolated, but also when the potential blood protein concentration was very high, as in the case of the whole blood experiments. In addition, they do not exhibit erythrotoxicity or leukocyte activation, and resist the adhesion of *E. coli*. The systematic increase of BMA moieties of up to 50% into the copolymers increased the hydrophobic properties of the coatings in the dry state but did not diminish the high hydrophilic properties in the wet state, i.e., the antiadhesive properties of the amphiphilic coatings were retained. Copolymers of SB and BMA can further reduce the self-assembly of the ionic groups and lead to a more rigid coating overall. In comparison to MPC- and CB-based polymers, SB moieties are less pH- and ion-responsive and not prone to degradation by hydrolysis. (Schönemann et al., 2018). In combination with the high degree of cyto-, hemo-, and immunocompatibility results the investigated class of amphiphilic sulfobetaine-based polymer coatings are promising candidates for blood-related biomedical applications. In this context, further studies are needed to transfer these coatings from model glass surfaces to flexible polyurethane and silicone rubber material surfaces used for blood catheters and indwelling catheters in the urogenital tract.

## Data Availability

The raw data supporting the conclusions of this article will be made available by the authors, without undue reservation.
