# Supplementary material for: In vitro biocompatibility analysis of protein-resistant amphiphilic polysulfobetaines as coatings for surgical implants in contact with complex body fluids
Source: Front Bioeng Biotechnol. 2024 Jul 17;12:1403654. doi: 10.3389/fbioe.2024.1403654 (PMC11288920; doi:10.3389/fbioe.2024.1403654)
Supplement: Supplementary file 1 [file DataSheet1.pdf]

Supporting Information

In vitro biocompatibility analysis of protein-resistant amphiphilic polysulfobetaines as coatings for surgical implants in contact with complex body fluids

Jana F. Karthäuser[1,§], Dierk Gruhn[2,6,§], Alejandro Martínez Guajardo[3], Regina Kopecz[1], Nina Babel[4], Ulrik Stervbo[4], André Laschewsky[3,5], Richard Viebahn[6], Jochen Salber[2,6*], Axel Rosenhahn[1*]

[1] Analytical Chemistry - Biointerfaces, Ruhr-University Bochum, Bochum, Germany

[2] Experimental Surgery, Ruhr-University Bochum, Bochum, Germany

[3] Institute of Chemistry, Universität Potsdam, Potsdam, Germany

[4] Centre for Translational Medicine, Medical Department I, Marien Hospital Herne, University Hospital of the Ruhr-University Bochum, Herne, Germany

[5] Fraunhofer Institute of Applied Polymer Research IAP, Potsdam, Germany

[6] Department of Surgery, Knappschaftskrankenhaus Bochum, University Hospital of the Ruhr-University Bochum, Bochum, Germany

Surface free energy determination

Table S 1: Surface tension σ_l with dispersive σ_l^d and polar σ_l^p part of testing solvents at 20 °C. [1–3]

Solvent	σ_l^d [mN/m]	σ_l^p [mN/m]	σ_l [mN/m]
Water	21.8	51.0	72.8
Glycerol	37.0	26.4	63.4
1-Bromnaphthalin	44.6	0.0	44.6

Surface energies were determined according to Owens, Wendt, Rabel, and Kaelble (OWRK).[1,2] Water, glycerol, and 1-bromnaphthalin droplets were applied on the coatings. The shapes of the droplets were captured using a CCD camera, and the contact angles between the liquids and the surrounding air on the surface were manually measured with the software ImageJ. Given the varying surface tensions of the liquids and their distinct polar σ_l^p and disperse σ_l^d fractions, the polar σ_s^p and disperse σ_s^d components of the coatings' surface free energy (SFE) can be calculated.

$$\frac{\sigma_l * (1 + cos(\theta))}{2\sqrt{\sigma_l^d}} = \sqrt{\sigma_s^d} + \sqrt{\sigma_s^p}\sqrt{\frac{\sigma_l^p}{\sigma_l^d}} \quad (1)$$

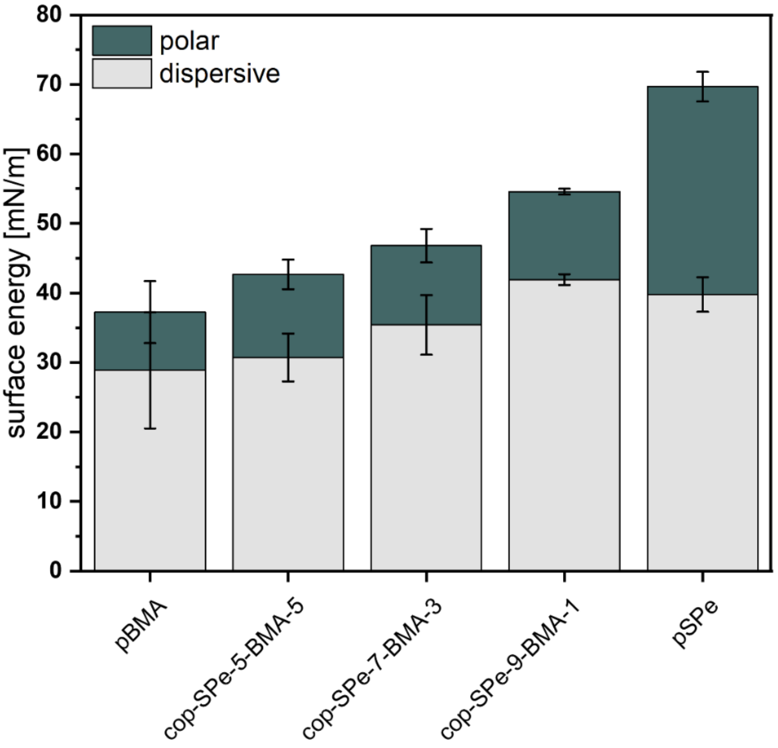

Figure S 1: Surface energy in the dry state of the coatings pBMA, cop-SPe-5-BMA-5, cop-SPe-7-BMA-3, cop-SPe-9-BMA-1, and pSPe with polar and dispersive components determined by OWRK method. [1,2]

L929 mouse fibroblasts adhesion assay

Table S 2: Surface area and number of cells for quantification of the L929 mouse fibroblasts adhesion assay on TCPS, PS, pBMA, cop-SPe-5-BMA-5, cop-SPe-7-BMA-3, cop-SPe-9-BMA-1, and pSPe surfaces.

Sample	Average of counted cells	Counted area [mm²]	Total area [mm²]	Total cells (calculated)	Comment
TCPS	107	0.2	113	60455	
PS	83	0.2	113	46895	
pBMA	76	0.2	113	42940	
cop-SPe-5-BMA-5	2	0.2	113	0	non adherent
cop-SPe-7-BMA-3	1	0.2	113	0	non adherent
cop-SPe-9-BMA-1	1	0.2	113	0	non adherent
pSPe	0	0.2	113	0	non adherent

(1) Owens, D. K.; Wendt, R. C. Estimation of the Surface Free Energy of Polymers. *J. Appl. Polym. Sci.* **1969**, *13* (8), 1741–1747. https://doi.org/10.1002/app.1969.070130815.

(2) Kaelble, D. H. Dispersion-Polar Surface Tension Properties of Organic Solids. *J. Adhes.* **1970**, *2* (2), 66–81. https://doi.org/10.1080/0021846708544582.

(3) Selvakumar, N.; Barshilia, H. C.; Rajam, K. S. Effect of Substrate Roughness on the Apparent Surface Free Energy of Sputter Deposited Superhydrophobic Polytetrafluoroethylene Coatings: A Comparison of Experimental Data with Different Theoretical Models. *J. Appl. Phys.* **2010**, *108* (1). https://doi.org/10.1063/1.3456165.
